# Civil conflict and sleeping sickness in Africa in general and Uganda in particular

**DOI:** 10.1186/1752-1505-1-6

**Published:** 2007-03-29

**Authors:** Lea Berrang Ford

**Affiliations:** 1 Deptartment of Population Medicine, University of Guelph, Canada; 2 233 Curzon Avenue, Saint Lambert, Quebec, J4P 2V3, Canada

## Abstract

Conflict and war have long been recognized as determinants of infectious disease risk. Re-emergence of epidemic sleeping sickness in sub-Saharan Africa since the 1970s has coincided with extensive civil conflict in affected regions. Sleeping sickness incidence has placed increasing pressure on the health resources of countries already burdened by malaria, HIV/AIDS, and tuberculosis. In areas of Sudan, the Democratic Republic of the Congo, and Angola, sleeping sickness occurs in epidemic proportions, and is the first or second greatest cause of mortality in some areas, ahead of HIV/AIDS. In Uganda, there is evidence of increasing spread and establishment of new foci in central districts. Conflict is an important determinant of sleeping sickness outbreaks, and has contributed to disease resurgence. This paper presents a review and characterization of the processes by which conflict has contributed to the occurrence of sleeping sickness in Africa. Conflict contributes to disease risk by affecting the transmission potential of sleeping sickness via economic impacts, degradation of health systems and services, internal displacement of populations, regional insecurity, and reduced access for humanitarian support. Particular focus is given to the case of sleeping sickness in south-eastern Uganda, where incidence increase is expected to continue. Disease intervention is constrained in regions with high insecurity; in these areas, political stabilization, localized deployment of health resources, increased administrative integration and national capacity are required to mitigate incidence. Conflict-related variables should be explicitly integrated into risk mapping and prioritization of targeted sleeping sickness research and mitigation initiatives.

## Background

**Figure 1:**
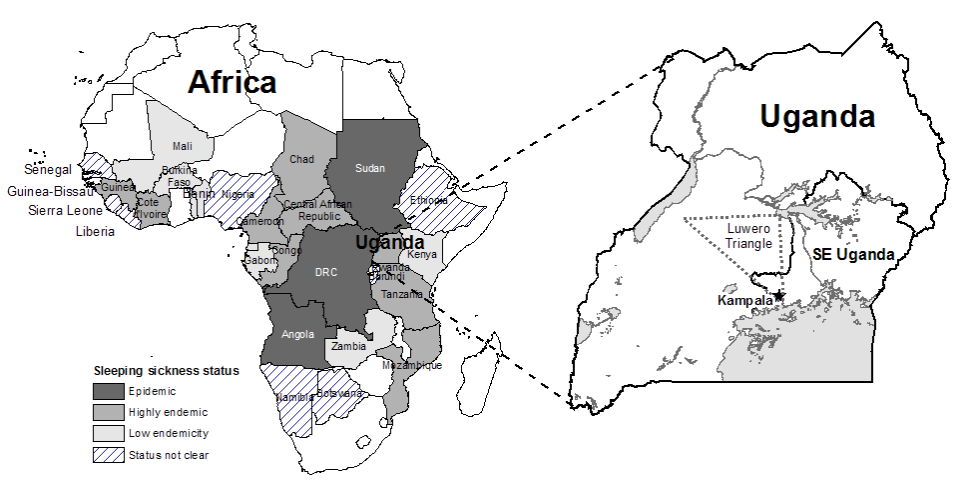
**Distribution of sleeping sickness in Africa and map of Uganda showing the case study area**. The approximate location of the 'Luwero Triangle' is shown; this is where much of the conflict and violence was concentrated during Uganda's civil war (1979–86). Sleeping sickness status data from WHO (2001).

Sleeping sickness re-emerged in Uganda in the 1970s, and continues to pose a public health and economic burden [1-3]. Similar re-emergence has been reported across sub-Saharan Africa since the 1970s, including outbreaks in the Democratic Republic of the Congo (DRC), Sudan [4], and Angola [5]. In many cases, sleeping sickness outbreaks have coincided with periods of civil conflict and instability in affected countries and regions. Conflict in this context refers to the occurrence of civil war, rebel insurgency, violent governance, political or military oppression of populations, and military combat. These temporal associations are not purely spurious; patterns and processes related to conflict have been identified as determinants of sleeping sickness incidence and outbreaks [4,5]. An improved understanding of the specific processes linking conflict to sleeping sickness incidence can guide geographical predictions of disease risk and optimization of intervention resources. This paper provides a review and characterization of the processes by which conflict has contributed to the occurrence of sleeping sickness outbreaks across sub-Saharan Africa, with a focus on south-eastern Uganda (Figure 1).

## Sleeping sickness: epidemiology, geographical distribution, and re-emergence

Sleeping sickness is the name used to describe the human form of African trypanosomiasis (*Trypanosoma spp*.), a protozoan parasitic disease affecting humans, livestock, and a large number of sylvatic species in much of sub-Saharan Africa (Figure 1). Transmitted by the tsetse fly vector (*Glossina spp*.), trypanosomiasis represents an important public health and economic burden in sub-Saharan Africa [6-8]. Sleeping sickness is characterized by highly variable and non-specific symptoms in its early stages [9], which are often mis-diagnosed as malaria [10]. Late stage sleeping sickness includes body weakness, progressive emaciation, slurred speech, mental confusion, and coma leading to death in all untreated cases [9]. There are two sub-species of human-infectious trypanosomes, including *T. b. gambiense*, which causes a more chronic disease, and is dominant in Western Africa, and *T. b. rhodesiense*, which causes more acute disease, and is generally found in East and Southern Africa, east of the Rift Valley [11]. The two forms of disease have different ecology, pathology, and epidemiology. *T. b. rhodesiense *progresses from early non-specific symptoms to infection of the central nervous system and death within months, while *T. b gambiense *typically follows a chronic clinical course progressing over several years.

Sleeping sickness treatment is expensive, complicated, and can be dangerous for the patient [12]. Current drugs are in scarce or uncertain supply, and there is limited optimism with respect to forthcoming drugs entering the market, particularly for the treatment of late-stage *T. b. rhodesiense *[12-17]. The dominant treatment for late-stage sleeping sickness that involves the central nervous system is melarsoprol, an organoarsenic compound with high toxicity and varying rates of treatment failure [12,18]. Active surveillance and case treatment have been found to be extremely effective in reducing disease transmission, particularly for *T. b. gambiense *[12,18], which is generally confined to a human-fly-human cycle [19]. *T. b. rhodesiense *transmission to humans is influenced by prevalence of the parasite in the animal reservoir; in east Africa, livestock represent an important reservoir for disease, and control of livestock infection and tsetse populations are important for reducing transmission to humans [12,19].

Sleeping sickness was first identified and characterized in Africa in the last few years of the 19^th ^century, a period that coincided with widespread and severe epidemics of the disease in Kenya, Tanzania, Uganda, Nigeria, and the Democratic Republic of the Congo (DRC). These epidemics have been associated with social and environmental disruptions during colonial administration [11,20], as well as livestock restocking following an 1889–1892 rinderpest epidemic [21]. The disease was generally brought under control by the 1960s in much of Africa but has re-emerged in many countries since the 1970s [8]. The re-emergence has been attributed to post-independence political turbulence, unstable governments, limited public health resources, and re-allocation of domestic and international funding towards malaria, HIV/AIDs, and tuberculosis.

In Uganda, a large epidemic of *T. b. rhodesiense *began in 1976 in the south-east of the country (Figure 1). Between 1976 and the decline of the epidemic in the mid-1990s, over 40,000 cases were reported in this region. Given estimates of significant under-reporting due to passive surveillance and diagnostic difficulties, the actual number of cases may have been ten times higher, with all unreported and untreated cases assumed to be fatal [3,22,23]. Though incidence declined in the 1990s, the disease continues to spread into new regions. Recent research suggests that central Ugandan districts may be at particularly high risk of infection and increased incidence [24]. These reports are particularly pertinent given that Uganda represents the boundary between the ranges of the two sub-species of sleeping sickness. While *T. b. rhodesiense *continues to spread from its traditional focus in the south and east, cases of *T. b. gambiense *continue to be recorded in north-western Uganda in the West Nile Region. These foci are currently separated by fewer than 200 kilometers, much of which is inhabited by tsetse flies. The two diseases differ widely in their treatment and control: it is very difficult to distinguish the two sub-species clinically [25]; a seriological card agglutination test (CATT) is commonly used for *T. b. gambiense *diagnosis, but is inappropriate for *T. b. rhodesience *– the test would not be effective in the presence of both diseases [26]; drug treatment regimes, which are expensive and can be dangerous, differ depending on the sub-species of infection [26]; approaches to control, such as focus on vector eradication, livestock treatment, or active livestock or human surveillance, will be more or less appropriate based on sub-species [25,26]; there may be potential for exchange of genetic material and increased drug resistance where the sub-species overlap [26,27]; cost-effective and successful control of sleeping sickness would become extremely difficult to achieve where both species were present. The potential for overlap of the two disease foci is therefore a considerable economic and public health concern [2].

Similar re-emergent outbreaks and incidence have been recorded in other countries (Figure 1). Angola experienced outbreaks in the late 1800s and early 1900s. By 1974, the year before Angola's independence, however, only three new cases of sleeping sickness were recorded [5]. Sleeping sickness re-emerged in Angola during a prolonged civil war following the country's independence in 1975 [19]. Although a peace agreement was signed in 2002, Angola's infrastructure and political situation remain highly unstable; the country is classified as 'epidemic' for sleeping sickness by the World Health Organization (WHO), along with the Democratic Republic of the Congo (DRC) and southern Sudan [28]. In Sudan, disease resurgence in the late 1970s was largely controlled by a Belgian-Sudanese trypanosomiasis treatment and control initiative [18]. Civil war in the 1980s and 1990s, however, lead to collapse of the control programme, and by 1997, sleeping sickness had re-emerged in Sudan with prevalence rates as high as 19% in south-western communities bordering the Democratic Republic of the Congo [18]. Re-emergence of disease and new epidemics were reported in the DRC in the 1970s and 1980s [29]. The DRC continues to experience Africa's highest burden of disease from sleeping sickness; in 1994, 72% prevalence was reported in one village, Kimbanzi [19]. The DRC represents an important source of infection for neighbouring countries. According to the WHO [28], Angola and southern Sudan have also reported high prevalences: between 20% and 50% in some communities. In several areas, sleeping sickness is the first or second greatest cause of mortality, ahead of HIV/AIDS [19]. Currently, the DRC, Angola, and Sudan remain the countries most affected by sleeping sickness.

Countries considered to be 'highly endemic', with sleeping sickness re-emergence and expected incidence increase include Cameroon, Uganda, Central African Republic, Chad, Congo, Côte d'Ivoire, Guinea, Mozambique, and Tanzania [28]. Incidence and prevalence are highly focal. In the Central African Republic, for example, villages bordering Sudan have reported epidemic levels of disease [18]. Sporadic or low endemic levels of sleeping sickness have been reported in Benin, Burkina Faso, Equatorial Guinea, Gabon, Kenya, Mali, Togo and Zambia. Unreported foci are likely to exist in additional countries, including Africa's most populated nation, Nigeria [30]. While few sleeping sickness cases have been reported in Kenya since the late 1960s and early 1970s, cattle infection remains an economic burden and there is ongoing incidence of disease across the border in south-eastern Uganda.

## Conflict, infectious disease, and sleeping sickness

Conflict and war have long been recognized as determinants of infectious disease risk [31,32]. Increased travel, trade, and inter-regional conflict have played a key role in the introduction and spread of diseases between civilisations and continents [33-35]. The influenza pandemic precipitated by World War I is a well-recognized example of a disease epidemic emerging from conflict conditions; deaths due to influenza exceeded deaths in battle [36]. Recent research in the Democratic Republic of the Congo (DRC) found that elevated mortality during the recent civil war was closely associated with violence and that most deaths were due to malnutrition or infectious disease [37]. On-going armed conflict has hampered the international polio eradication campaign, particularly in Somalia and Afghanistan, areas of persistent polio infection and high insecurity [38,39]. Outbreaks and increased disease incidence have been attributed to a range of factors associated with conflict [40], including decreased hygiene, dietary deficiencies, decline of health services, travel insecurity, reduced access of humanitarian support, reduced veterinary and zoonoses control, and internal displacement of populations into marginal areas. The associations between conflict and infectious disease are particularly prevalent in Africa, where there remain many foci of ongoing civil conflict, and where infectious diseases remain important contributors to national mortality [41-43].

The sensitivity of sleeping sickness incidence to conflict-related processes is associated with the low transmission potential of the disease. A general model of the disease was developed by Rogers [44]. While developed for animal trypanosomiasis, an adapted and simplified equation can provide useful insights into the parameters affected the transmission potential of sleeping sickness; here we adapt the model for one tsetse species, one trypanosome species, and one host species – in this case we are interested in human disease, but animals are also important reservoirs of infection, particularly in the case of *T. b. rhodesiense*. The reproductive rate (R_0_) can be estimated using the following general equation [44-46]:

R_0 _= *α*^2^*mbce*^-*u*T^/*ur *    (Eq. 1)

Where:

*R*_0 _Basic reproductive ratio

*α *Daily biting rate of flies on humans (or animal reservoirs)

*m *Ratio of tsetse flies to humans (or animals)

*b *Probability of a fly becoming infected from an infected person (or animal)

*c *Probability of a person (or animal) becoming infected from an infected fly

1/*u *Life expectancy of tsetse flies (days)

*T *Incubation period in tsetse flies (days)

1/*r *Duration of infection in a person (or animal) (days)

R_0 _reflects the number of additional cases that a single case is expected to generate and therefore represents the transmission potential of a pathogen; transmission will occur and epidemics can result when R_0 _> 1. The values of the parameters vary based on the species of host, parasite and vector of interest, as well as the context of the area and situation. In this case, we assume that the values of *b*, *c*, and *T *are constant and unrelated to political or social events. The remaining parameters, *m*, *α*, 1/*u*, and 1/*r*, however, could vary due to external influences.

In humans, the R_0 _value is generally below one and transmission cannot sustain itself; *T. b. rhodesiense *is mainly a zoonosis, with disease occurring commonly in cattle populations. In humans, periods of relatively low-level or undetected disease are punctuated by periods of severe outbreak [27]. Sleeping sickness, particularly *T. b. rhodesiense*, is characterized by the occurrence of distinct epidemics whose temporal occurrence has been consistently observed to parallel or lag periods of conflict in affected areas [24,47]. This epidemic pattern is a function of the low transmission potential of sleeping sickness compared to other infectious diseases; one case of sleeping sickness will be likely to result in further cases only under particular circumstances strongly favouring transmission [8]. Transmission can be enhanced by increasing the equation parameters (Eq. 1) [8]; when sufficiently strong or prolonged, an outbreak is triggered. Conflict-related processes, therefore, can influence sleeping sickness incidence by affecting the parameters in the model (*m*, *α*, 1/*u*, or 1/*r*); these can include a range of ecological, social, and biophysical determinants [1]. Impacts on the biting rate of flies (*α*), such as increased exposure of people to infested areas, will be particularly important as this parameter exponentially affects the value of R_0_. Similarly, conditions affecting the lifespan of the fly (1/*u*), such as vector control measures, will be important as this parameter appears twice in the equation.

## The case of south-eastern Uganda

**Figure 2:**
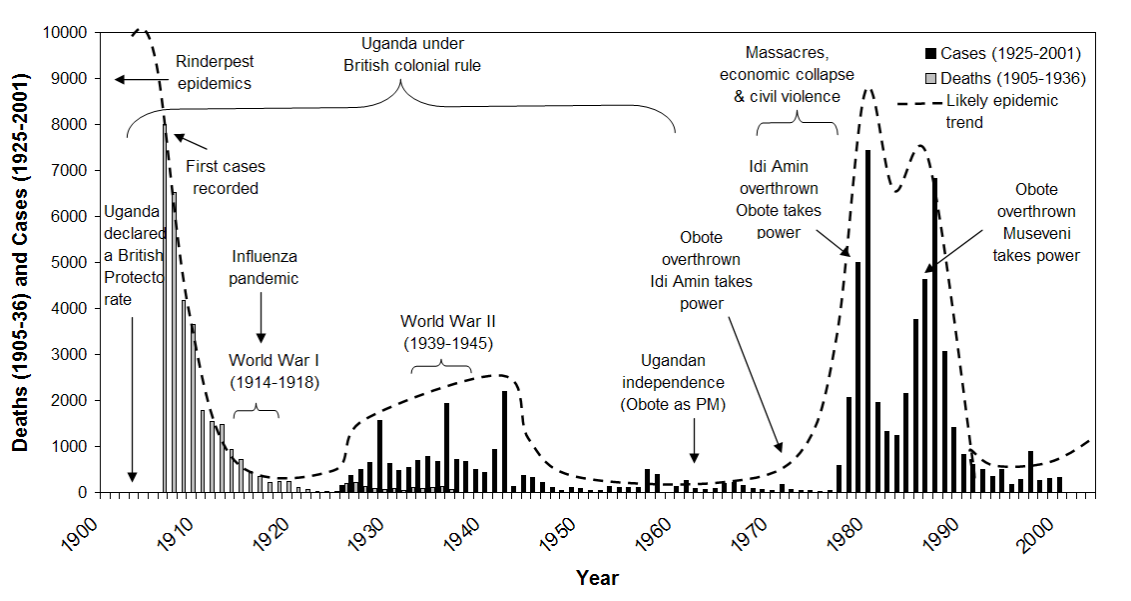
**Sleeping sickness epidemics and major political events in Uganda, 1905–2000**. Cases from 1936 onwards include south-eastern Uganda only. *Sources: *Sleeping sickness data 1905–36 deaths [62], 1925–36 cases [62], 1937–58 cases [63], 1960–71 cases (Unpublished report, 1992, Mbulamberi, D. B. The sleeping sickness situation in Uganda: past and present. National Sleeping Sickness Control Program, Jinja, Uganda), 1972–75 cases [54], and 1976–2001 cases (Ministry of Health, Uganda); Political time-series [56, 64, 65].

Figure 2 shows a time-line of sleeping sickness incidence in south-eastern Uganda between 1900 and 2000. As seen in this figure, the epidemic of 1900–1920 coincides with establishment of colonial rule in Uganda, while the 1940–1946 epidemic coincides with World War II. There has been extensive discussion of the role of colonial governance in the 1900–1920 sleeping sickness outbreak [11,21,27,48-51]. The more recent *T. b. rhodesiense *epidemic in 1976–1990s coincided with political instability and civil war during and after the rule of Idi Amin [24]. Uganda's civil war influenced the transmission potential of sleeping sickness in a number of ways, including: breakdown of veterinary and public health services (↑1/*r*) [52-54]; collapse of vector control (↑*m*, ↑1/*u*); regrowth of bushy tsetse habitat in abandoned agricultural fields (↑*m*) [19,52]; increasing displacement of human and animal populations into marginal or swampy areas where they are more likely to be bitten by flies (↑*α*) [52]; In Uganda, internally displaced people (IDP) fled areas of intense conflict, particularly in the 'Luwero Triangle' region (Figure 1), an area in south-central Uganda where much of the conflict and violence was concentrated [55,56]. Refugees and IDPs returning home after the conflict faced increased risk due to the vegetation and new tsetse habitat that had grown during their absence (↑*m*, ↑*α*). These processes directly affected a number of parameters in the sleeping sickness transmission model (*m*, *α*, 1/*u*, 1/*r*), increasing the transmission potential of the disease (R_0_). The R_0 _value increased via these processes over several years (resulting in a time lag between the peak in civil conflict and the outbreak peak) before exceeding the threshold required for an outbreak.

## Conflict and sleeping sickness in sub-Saharan Africa

The trends and processes contributing to south-eastern Uganda's sleeping sickness epidemic have also been observed in other countries. Despite the epidemiological differences between *T. b. rhodesiense *and *T. b. gambiense*, the temporal occurrence of outbreaks or high incidence of both sub-species are associated with times of conflict. An association between land cover change and sleeping sickness similar to the process observed in south-eastern Uganda has been suggested in Kenya; increased vegetation growth around homesteads and the resulting movement of tsetse flies into peridomestic environments likely contributed to the sleeping sickness outbreaks in Kenya in 1965 [57]. Outbreaks in the 1990s in Sudan, as in south-eastern Uganda, have been linked to abandonment of land, bush invasion, and increased risk of exposure for returning IDPs [52]. In north-western Uganda in the West Nile region, *T. b. gambiense *infection is believed to have been introduced to the area by refugees returning from infected areas of Sudan following Uganda's civil war [58].

Collapse of essential health services, and veterinary and vector control contribute consistently to increased disease risk through impacts on the transmission parameters of sleeping sickness (*m*, 1/*r*, and 1/*u*). Reduced surveillance and treatment directly affect the duration of infection in both humans and animal reservoirs of infection (↑1/*r*), while reduction of health and veterinary services increase the duration of infection in people and infected animals (↑1/r). Collapse of vector control can affected both tsetse numbers (↑*m*) and the average lifespan of flies in the area (↑1/*u*). Surveillance and treatment are particularly important for the mitigation and control of *T. b. gambiense*. In Sudan, for example, absence of active case finding was a major factor contributing to the resurgence of sleeping sickness in the 1990s [18]. Sudanese health and disease control infrastructure was essentially non-operable during two decades of civil war in the 1970s-1990s [19]. In the DRC, negligible staff salaries, lack of motivation, poor road conditions, petrol shortages, and corruption were identified as key constraints to the efficacy of mobile surveillance teams, which failed to contain sleeping sickness incidence in the 1990s [19]. Limited or absent veterinary and vector control programs can be more important in *T. b. rhodesiense *areas, where livestock reservoirs can have considerable influence on transmission potential [21,59]. Insecurity due to conflict further constrains the capacity of both national governments and external organizations to respond to outbreak situations. In Angola, for example, peripheral mining regions were subject to active insurgencies, resulting in high insecurity; this made implementation of sleeping sickness control activities logistically impossible [60]. Collapse of training schools for health care workers, nurses and laboratory technicians contribute to further decline in sleeping sickness control initiatives (↑ 1/*r*). Abel et al. [60] note that in the 1990s, at least two Angolan diagnostic and treatment centres had to be abandoned due to rebel attacks.

In many war-zone outbreaks, non-governmental organizations (NGO) and United Nations (UN) agencies assume partial or full responsibility for outbreak response [5,18]. In Angola in the 1990s, for example, religious organizations played an important role in intervention activities when high insecurity in the country limited UN and international NGO intervention in remote areas [60]. While external humanitarian support may contribute essential services, lack of harmonization and integration of activities between organizations is a constraint to consistent and continuous sleeping sickness control during conflict [18,19]. In some cases, resurgence of sleeping sickness during periods of conflict has occurred in previous disease hot spots, as in the case of Sudan [18]; in others, resurgence is occurring outside of traditional disease foci, as is currently occurring in sleeping sickness spread into central Uganda [24].

The range of specific processes by which conflict has contributed to sleeping sickness risk is summarized in Table 1. This summary identifies the dominant impacts and vulnerabilities associated with conflict, their effects on the transmission determinants of sleeping sickness, and the resulting impact on the R_0 _equation parameters of sleeping sickness. Four key impact categories are identified, including: 1) Economic and global effects, 2) Decline of health systems and services, 3) Forced migration and internal displacement of populations, and 4) Regional insecurity and restricted access for external humanitarian support.

**Table 1:** Impacts of social conflict on sleeping sickness in sub-Saharan Africa

**Impacts and vulnerabilities associated with conflict**	**Impact on transmission determinants**	**Parameter impact**
**Economic and global effects**		
		
• Reliance of governments on external funding for control resources and donor reluctance due to political insecurity	Reduction in provision of public health services	↑ 1/*r *in people
• Reduced reliability of economy for international investment and aid		
• Collapse of businesses and local economies. Loss of employment, income &provision of products & services	Decreased treatment and control of animal infection and transmission	↑ 1/*r *in animal reservoirs
• Abandonment or appropriation of land; changes in land ownership and land use. Possible increased vector habitat		
• Loss of educated and business elite in cases of emigration, exile, or purging; decreased overall response capacity and economic stability	Decreased vector control	↑ 1/*u*, ↑*m*
		

**Decline of health systems and services**		
• Absence of public health funding due to economic collapse, corruption, or re-allocation of funds to security or military expenses	Reduction in provision of public health services	↑ 1/*r *in people
• Collapse or decline in training programs for public health, veterinary, and vector control workers; decline in personnel and expertise; limited surge capacity for outbreak response	Decreased treatment and control of animal infection and transmission	↑ 1/*r *in animal reservoirs
• Total absence of case surveillance or reporting in some rebel-controlled areas or high-conflict zones; limited screening/treatment		
• Demotivation of health care, veterinary and vector control personnel caused by insecurity, inflation, or no pay	Decreased vector control	↑ 1/*u*, ↑*m*
• Collapse of vector control and veterinary health programs		
		

**Forced migration and internal displacement of populations**		
• Increased mortality and morbidity due to conflict violence	Increased exposure of people and cattle to tsetse habitat	↑ *a*
• Transport of people and animal hosts, vectors, and parasites into potentially naïve or uninfected populations		
• Loss of livelihoods – increased stress, reduced household resources, reduced health &nutritional status	Increased vector habitat	↑*m*
• Decreased access to health facilities; decreased population health	Increased mortality	-
• Separation of household units		
• Abandonment of land; vegetation re-growth; increased vector habitat		
		

**Regional insecurity and restricted access for external humanitarian support**		
•Treatment facilities prone to looting and insurgent attacks	Reduction in provision of public health services	↑ 1/*r *in people
• Insecurity in affected regions constrains or prevents control and implementation logistics; access of mobile teams limited	Increased mortality	-
• Lack of integration and continuity in primary care where a range of NGOs are the dominant providers of health and intervention services.		
• Patients delay seeking medical help due to travel insecurity or unavailable transport; higher proportion of late-stage or unreported and untreated cases.		

The proportionate importance of the factors summarized in Table 1 will differ between *rhodesiense *and *gambiense *affected areas. Transmission during g *ambiense *outbreaks is usually dominated by a human-fly-human cycle. Reduced treatment of humans and decline of health systems will therefore have greater impact in *gambiense *affected regions. In these areas, decline of health services leads to increased duration of human infection (↑1/*r *in humans). Changes in livestock infection will have greater impact in *rhodesiense *affected regions. In these areas, civil war leads to reduced cattle treatment, thus increasing the duration of cattle infection (↑1/*r *in cattle) and the reservoir of human-infectious parasites for transmission to the human population. In both *gambiense *and *rhodesiense *affected regions, reduced tsetse control increases vector populations (↑*m*, ↑1/*u*). Displacement of people and animals into marginal, bushy or swampy areas further promotes increased human, fly, and cattle contact (↑*a*).

## Discussion

Conflict is a major determinant of sleeping sickness outbreaks in sub-Saharan Africa. Efforts to prevent and control sleeping sickness must identify and integrate knowledge of the processes by which conflict affects disease risk. Prioritization of high-risk areas and targeted intervention can be optimized by consideration of conflict in sleeping sickness-affected countries. Sleeping sickness intervention in Africa is constrained by a range of factors and processes. Increased drug development is needed to identify and develop newer, safer drugs with more secure availability and supply to the African market. The epidemic nature of sleeping sickness means that when cases decline, resources are often rapidly reallocated to other health priorities until the occurrence of another outbreak. Consistent and active surveillance is difficult to rationalize during inter-outbreak periods in affected countries where resources are strained. Since late-stage sleeping sickness cases are expensive and difficult to treat, however, active surveillance, early treatment, and outbreak prevention can considerably reduce the burden of disease. In countries recovering from recent civil war, rapid re-establishment of essential health services and active surveillance and treatment will be central to reducing sleeping sickness incidence. The focal nature of sleeping sickness means that resources can be optimized in the short term by targeting outbreak locations and areas bordering countries with high incidence. In cases such as Uganda, where conflict is intermittent and regional, intervention is not extensively constrained by insecurity. Mobilization of national and international resources to support intervention in central Uganda is logistically feasible. As noted by Fèvre et al. [2], *T. b. rhodesiense *is theoretically not difficult to prevent, but can be challenging to control once established. In the case of *T. b. rhodesiense *in Uganda, surveillance and control of livestock infection/movements can contribute to a decrease in both the animal reservoir of infection and spread of the parasite to new populations. Much of this support will need to be targeted at policy and infrastructure development. Central Uganda is currently experiencing spread of disease and establishment of new *T. b. rhodesiense *foci; rapid intervention to curb these developments is needed to prevent increased burden of disease due to sleeping sickness in Uganda.

Conflict has played an important role in contributing to the incidence and distribution of sleeping sickness in sub-Saharan Africa. Regrettably, the causes of sleeping sickness are also the main constraints to eradication initiatives: "virtually by definition, [sleeping sickness] is a public health problem in places where a research infrastructure can hardly exist" [[60] p. 147]. The campaign to eliminate the tsetse vector from the African continent [12,14,61] will face enormous constraints due to continued conflict. Absence of appropriate administrative infrastructures for program implementation in affected countries represents the most acute challenge to such campaigns; this is true even for countries such as Uganda where conflict is only intermittent or regional. In Uganda, administrative capacity and intersectoral cooperation are important constraints to coordination of intervention activities [2]. While top-down, continent-wide eradication campaigns are ambitious and appealingly goal-oriented, progress to curb sleeping sickness is more likely to come from slow development of national capacity, policy infrastructure, administrative integration and political stabilization in affected countries. In many cases, insecurity due to conflict has constrained international or external intervention and control. Local interventions, with localized infrastructure and rural deployment capacity, may be better placed to provide essential services during times of intense or widespread conflict. Donors and aid agencies should continue to support national, regional, and community mitigation and intervention initiatives towards the common goal of reducing and eliminating sleeping sickness burden.

Prioritization of high-risk areas for sleeping sickness and trypanosomiasis control should explicitly integrate the occurrence of conflict and its impacts on transmission risk. The occurrence of conflict and presence of large number of internally displaced people can be integrated into current risk maps in addition to land cover, tsetse habitat, and livestock distributions. An understanding of areas where conflict may contribute to increased disease risk can guide prioritization of continent-wide as well as national mitigation programs.

## Conclusion

Conflict is an important determinant of sleeping sickness outbreaks in sub-Saharan Africa. In Uganda, the two sub-species of sleeping sickness can be expected to merge in the absence of immediate and targeted intervention in central districts; the presence of both diseases in one region will dramatically increase burden of disease as well as the complexity and difficultly of subsequent control initiatives. Control and prevention of sleeping sickness by national and international authorities should explicitly integrate consideration of conflict and its impacts into mapping and targeting of regions for priority intervention. Prevention and control campaigns should be assessed and evaluated against the ability of the initiative to address, mitigate, or alleviate the conflict-related drivers of disease risk. The occurrence of sleeping sickness in conflict-affected areas will severely constrain the success and cost-benefit evaluations of continent-wide tsetse eradication campaigns. Prevention of sleeping sickness risk in affected sub-Saharan African countries requires increased international focus on development of administrative policy, capacity, integration, and infrastructure to implement localized control strategies.

## Abbreviations

HIV/AIDS Human Immuno-deficiency Virus/Acquired Immuno-deficiency Virus

DRC Democratic Republic of the Congo

WHO World Health Organization

IDP Internally Displaced Persons

## Competing interests

The author(s) declare that they have no competing interests.

## Authors' contributions

LBF conceived, designed, researched, and prepared the manuscript.
